# Tissue turnover and rejuvenation through mechanics

**DOI:** 10.3389/fcell.2026.1828482

**Published:** 2026-06-17

**Authors:** Mizuki Uchida, Yusuke Toyama

**Affiliations:** 1 Mechanobiology Institute, National University of Singapore, Singapore, Singapore; 2 Department of Pharmacology, Graduate School of Pharmaceutical Sciences, Hokkaido University, Sapporo, Japan; 3 Department of Biological Sciences, National University of Singapore, Singapore, Singapore

**Keywords:** apoptosis, cell extrusion, cellular rejuvenation, cellular senescence, mechanobiology, senolytics, senomorphics, tissue homeostasis

## Abstract

Tissue turnover depends on the efficient removal of damaged cells to maintain structural and functional integrity. Cells respond to damage across a spectrum of outcomes, ranging from repair and recovery to senescence, apoptosis, and malignant transformation. This review focuses on apoptosis and senescence in epithelia as two outcomes with profound consequences for tissue homeostasis, one ensuring swift mechanical removal of damaged cells and the other driving persistent tissue disruption. While apoptotic cells are efficiently eliminated through coordinated mechanical processes, senescent cells resist elimination and progressively impair tissue function through chronic inflammation and mechanical remodeling of the tissue microenvironment. Restoring tissue function therefore requires strategies to efficiently clear or reverse the senescent state. Immune-mediated clearance and senolytic drugs can reduce senescent cell burden, yet their efficacy and selectivity remain limited. Emerging mechanobiological strategies, including low-frequency ultrasound and geometric confinement, offer a complementary approach by directly reversing the senescent phenotype through physical cues. Ultimately, harnessing these mechanical forces offers a promising avenue toward restoring tissue function.

## 1 Introduction

**FIGURE 1:**
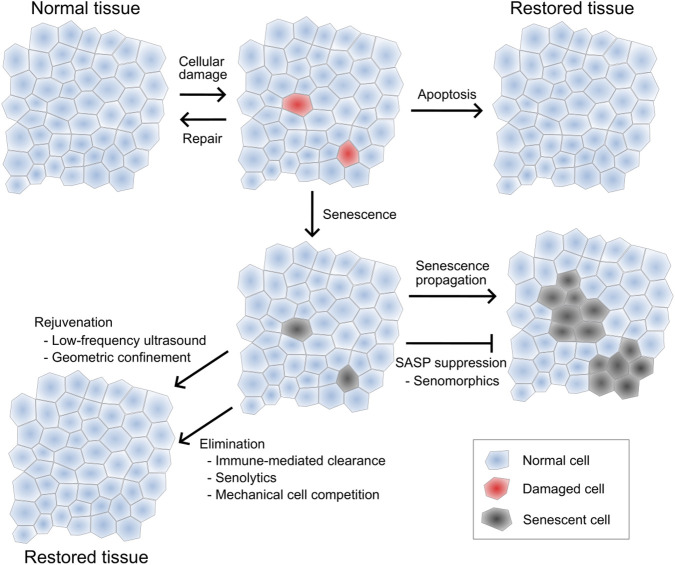
Overview of tissue turnover through apoptosis and cellular senescence. Damaged cells within a tissue undergo either repair or apoptotic cell removal (Section 2), restoring tissue homeostasis. Alternatively, damaged cells may enter a state of cellular senescence (Section 3), characterised by resistance to elimination. Senescent cells propagate the senescent state to neighbouring cells through paracrine SASP signalling and mechanical remodelling of the tissue microenvironment (Section 3). To restore tissue homeostasis, senescent cells can be eliminated through immune-mediated clearance, senolytics, or mechanical cell competition (Section 4). Alternatively, the pathological output of senescent cells can be suppressed by senomorphics, which target the SASP without inducing cell death (Section 4). The senescent phenotype can also be reversed through mechanobiological rejuvenation strategies, including low-frequency ultrasound and geometric confinement (Section 4).

Cells are constantly exposed to a variety of stresses, including oxidative damage, radiation, mechanical stress, and replication errors (Ciccia and Elledge, 2010). The severity and persistence of damage determines the outcome of this graded cellular response: mild damage activates DNA repair pathways, allowing the cell to return to normal; moderate or persistent damage triggers a permanent exit from the cell cycle known as cellular senescence; severe and irreparable damage initiates programmed cell death through apoptosis; and when these checkpoint mechanisms fail, uncontrolled proliferation leads to cancer (Jackson and Bartek, 2009). This review examines two of these outcomes, apoptosis and cellular senescence, through a mechanobiological lens, as mechanical forces are known to influence cell fate and both outcomes have profound mechanical dimensions that are intimately linked to tissue homeostasis. Apoptotic cells are efficiently eliminated from tissue through a well-coordinated mechanical process in which neighboring cells drive their physical removal (Arnould et al., 2025). Senescent cells, by contrast, resist elimination, persist within the tissue, and progressively disrupt the microenvironment through the secretion of pro-inflammatory and catabolic factors (Campisi and d’Adda di Fagagna, 2007). We first examine the mechanical mechanisms of apoptotic cell removal, then explore how senescent cells evade these processes, and finally discuss emerging strategies to counteract the senescent state (Figure 1).

## 2 Elimination of apoptotic cells

Apoptosis is a form of programmed cell death that plays an essential role in development, tissue homeostasis, and the removal of damaged or potentially dangerous cells (Taylor et al., 2008). Both intrinsic and extrinsic pathways converge on the activation of caspases, a family of cysteine proteases that execute the dismantling of the cell. The Bcl-2 family of proteins acts as a critical regulatory switch, with pro-apoptotic members (Bax, Bak) promoting and anti-apoptotic members (Bcl-2, Bcl-xL) inhibiting the release of cytochrome c from the mitochondria and subsequent caspase activation. The execution of apoptosis is accompanied by a set of characteristic morphological changes, including apoptotic volume decrease, a rapid loss of cell volume driven by ion efflux, membrane blebbing, chromatin condensation, and fragmentation of the cell into membrane-bound apoptotic bodies that are subsequently cleared by phagocytes. This section focuses on the mechanical aspects of apoptotic cell removal, particularly in epithelial tissues, where the physical process of extrusion has been extensively studied.

### 2.1 Epithelial cell extrusion

The physical removal of apoptotic cells from epithelial tissue occurs through a process known as cell extrusion (Rosenblatt et al., 2001), where the dying cell is squeezed out of the monolayer while maintaining epithelial barrier integrity. Cell extrusion requires orchestrated mechanical forces within both the apoptotic cell and its neighbors. Upon caspase activation, significant morphological changes take place within the dying cell. Many of these are caspase-dependent, as these proteases cleave a wide array of target proteins (Fischer et al., 2003). For instance, ROCK1 (Rho-associated protein kinase 1) is cleaved into a constitutively active form, which then phosphorylates the myosin light chain to drive actomyosin contraction within the dying cell, contributing to membrane blebbing and apoptotic body formation. In parallel, caspase-mediated cleavage of MRCKα (myotonic dystrophy kinase-related Cdc42-binding kinase-α) generates a constitutively active fragment required for assembly of an actomyosin ring within the dying cell that drives extrusion (Gagliardi et al., 2018). Simultaneously, caspase activity promotes the accumulation of S1P (sphingosine-1-phosphate), a bioactive lipid released by the apoptotic cell. S1P acts as a paracrine signal, binding to S1P_2_ receptors on the membranes of adjacent epithelial cells (Gu et al., 2011). This binding triggers Rho-mediated actomyosin contraction in the neighbors, leading to the assembly of a supracellular actomyosin purse-string within the neighboring cells. The actin-binding protein Coronin 1B contributes to force-dependent remodeling of the cortical actin network, facilitating the assembly of the supracellular actomyosin ring (Michael et al., 2016). Moreover, the unconventional motor myosin VI mediates the transmission of mechanical tension at adherens junctions and is required for the activation of Rho signaling in cells adjacent to the dying cell (Duszyc et al., 2021).

The contraction of this ring, coupled with basal drifting directed by microtubules, generates centripetal forces that constrict around the dying cell, pushing it out of the epithelial plane (typically in the apical direction in mammalian cells) (Slattum et al., 2009). This process is accompanied by the loosening of the cell’s adhesion to both neighboring cells and the ECM (extracellular matrix), associated with caspase-dependent cleavage of cell adhesion proteins, including E-cadherin and integrins (Kocgozlu et al., 2016; Teng et al., 2017). As the apoptotic cell progressively loses its attachments, reducing resistance to extrusion, the neighboring cells fill the space beneath it (Kocgozlu et al., 2016; Balasubramaniam et al., 2025), displacing the dying cell upward and closing the gap (Levayer, 2024). Sealing this gap requires the *de novo* formation of cell-cell junctions, ranging from tight junctions to desmosomes, between the converging neighbors underneath the apoptotic cell. The precise mechanisms governing this process remain poorly understood, and whether these junctions form *via* a ‘zippering’ mechanism that seals the epithelium and pushes the dying cell out remains a key area for further investigation.

### 2.2 Mechanical impact of apoptosis on neighboring cells

Beyond its role in physically expelling the dying cell, the extrusion process generates mechanical signals that propagate into and reshape the surrounding tissue. Actomyosin contraction within the dying cell and the assembly of the supracellular actomyosin cable in neighboring cells generate centripetal forces. The resulting forces stretch the immediate neighbors and propagate through the tissue *via* cell-cell adhesions. Consequently, forces associated with apoptosis, apoptotic forces (Teng and Toyama, 2011), drive localized cellular responses and guide larger morphogenetic changes.

The stretching of neighboring cells activates Piezo1, a mechanically activated ion channel in the plasma membrane, which transduces mechanical strain into intracellular signals. This Piezo1 activation leads to Ca^2+^ influx (Gudipaty, 2017), which propagates as Ca^2+^ waves through mechanosensitive channels to surrounding cells (Yamada, 2025). Gap junctions, IP3 (inositol 1,4,5-trisphosphate) receptors, and the mechanosensitive channel TRPC1 (transient receptor potential canonical 1) were identified as additional mediators of Ca^2+^ wave propagation during cell extrusion (Takeuchi et al., 2020). These waves drive Rac-mediated collective migration and support the actomyosin remodeling required to seal the gap left by the apoptotic cell (Cho et al., 2024). In parallel to calcium signaling, ERK (extracellular signal-regulated kinase) activity propagates outward from extruding cells, promoting survival and coordinating collective movement in the surrounding epithelium (Gagliardi et al., 2021; Valon et al., 2021). Furthermore, cell stretching of the neighboring cells leads to the rearrangement of the cytoskeleton, including the stretching of the intermediate filament network, particularly cytokeratin-18 (Thomas et al., 2020). This elevated tension in the intermediate filaments could propagate to the nucleus *via* the LINC (linker of nucleoskeleton and cytoskeleton) complex, causing nuclear compression or flattening and triggering downstream mechanotransduction, including YAP (yes-associated protein) nuclear translocation (Koushki et al., 2023).

Mechanotransduction in neighboring cells has been shown to define their subsequent fate. Recent studies indicate that YAP translocates from the cytoplasm to the nucleus, initiating cell cycle progression and leading to apoptosis-induced compensatory proliferation (Kawaue et al., 2023). Importantly, YAP nuclear translocation is spatially inhomogeneous, occurring only in neighboring cells that experience high strain and possess larger nuclei with a higher number of nuclear pore complexes. This spatial selectivity is a critical regulatory feature, preventing the over-proliferation of neighboring cells. The mechanical role of apoptotic forces in compensatory proliferation complements the caspase-dependent release of mitogenic signals, which stimulate the division of surrounding cells.

Apoptotic forces also drive macroscopic tissue remodeling. For example, during *Drosophila* dorsal closure, cell contraction and elimination generate contractile forces that pull the neighboring epidermis together (Toyama et al., 2008). Furthermore, dying cells can act as mechanical drivers of tissue folding. The transient apico-basal forces generated during apoptosis are sufficient to deform the apical surface. This process involves the formation of an apico-basal actomyosin cable that anchors to the nucleus. As this cable contracts, it exerts tension that eventually drives progressive epithelial bending, as seen in the formation of *Drosophila* leg joints (Monier et al., 2015) and the bending of the avian neural tube (Roellig et al., 2022).

## 3 Characteristics and persistence of senescent cells

In contrast to apoptotic cells, which are removed through these coordinated mechanical processes, senescent cells adopt a fundamentally different fate, one of persistence rather than elimination. Cellular senescence is a state of stable cell cycle arrest triggered by genotoxic stressors such as telomere shortening, irradiation, and oxidative stress. Mechanical stimuli, including substrate stiffness, have also been shown to contribute to cellular senescence (Jaalouk and Lammerding, 2009). To prevent the propagation of damaged cells, tumor suppressors like p53 and p16 enforce this permanent exit from the cell cycle through the p21 and Retinoblastoma (Rb) pathways (Engeland, 2022). Unlike apoptotic cells, senescent cells remain metabolically active and secrete a SASP (senescence-associated secretory phenotype), involving the release of cytokines, growth factors, and MMPs (matrix metalloproteinases) that drive chronic inflammation and promote senescence in neighboring cells, a process known as paracrine senescence (Coppé et al., 2010). Phenotypically, these cells are characterized by an enlarged, flattened morphology, increased β-galactosidase activity, and a disturbed nuclear architecture, often involving the loss of Lamin B1, which compromises nuclear stiffness (Gorgoulis et al., 2019).

Cellular senescence is not inherently detrimental. Transient senescence plays beneficial roles during embryonic development, where programmed senescent cells contribute to tissue patterning and remodeling (Muñoz-Espín et al., 2013; Storer et al., 2013), and during wound healing, where senescent fibroblasts and endothelial cells limit fibrosis and promote tissue repair through SASP-mediated recruitment of immune cells (Demaria et al., 2014). In these physiological contexts, senescent cells are efficiently cleared by the immune system once their function is complete. The pathological problem therefore lies not with senescence itself, but with the chronic accumulation of senescent cells that escape clearance in aged or damaged tissues. This distinction poses a key therapeutic challenge: interventions must selectively target the persistent, tissue-disrupting senescent population, or their pathological output, while conserving the transient, beneficial senescent cells.

### 3.1 The mechanobiology of senescence

Senescent cells are known to remodel their mechanical environment to reinforce their own persistence, creating a progressively stiffer and more dysfunctional tissue niche (Bajpai et al., 2021). The progression of senescence is deeply intertwined with structural alterations in the ECM and the cytoskeleton. With age, the ECM undergoes remodeling and increased collagen cross-linking, which leads to tissue stiffening. Senescent cells sense this change through focal adhesion proteins (Starich et al., 2024), and develop a highly polymerized and stiffened actin network (Rebehn et al., 2023). Collectively, these cytoskeletal changes reduce cell motility and adaptability while increasing cell rigidity. These mechanical shifts influence the nucleus, altering mechanotransduction and structural integrity. In normal contexts, ECM stiffening promotes YAP/TAZ (transcriptional coactivator with PDZ-binding motif) nuclear translocation, driving proliferation and tissue growth. However, chronic stiffening in aged tissues dysregulates this mechanosensory response, with aberrant YAP/TAZ signaling contributing to fibrosis and reinforcing the senescent microenvironment (Liu et al., 2015; Joung et al., 2025). Furthermore, altered mechanical cues lead to defects in the nuclear lamina and chromatin reorganization. These defects disrupt DNA repair mechanisms and lock the cell into a permanent growth-arrested state. Nuclear envelope rupture, a mechanical consequence of Lamin B1 loss, further activates the cGAS-STING (cyclic GMP-AMP synthase/stimulator of interferon genes) pathway, amplifying SASP and reinforcing the senescent state (Dou et al., 2017; Sladitschek-Martens et al., 2022). In this manner, senescence propagates through a tissue not only *via* soluble SASP factors but also through the altered mechanical landscape they create.

Beyond propagating the senescent state itself, the mechanical and secretory output of senescent cells can also impair the surveillance mechanisms by which the surrounding non-senescent epithelium clears damaged or transformed cells. Senescent cells secrete HGF (hepatocyte growth factor) as part of the SASP, which inhibits the apical extrusion of oncogenic RasV12-expressing cells in a cell competition assay and promotes the survival of mutated cells in mouse liver and intestine (Igarashi et al., 2022). Senescence-associated stiffening of the tissue microenvironment may further compromise extrusion, since elevated baseline tension at adherens junctions has been shown to impair the apical extrusion of apoptotic cells in epithelial monolayers (Mann et al., 2024). Together, these findings suggest that senescent cells not only disrupt their own tissue mechanically but also compromise the function of the surrounding non-senescent epithelium.

### 3.2 Mechanisms of survival and evasion of senescent cells

A primary reason senescent cells persist is their acquired resistance to apoptosis. Despite significant damage, these cells upregulate pro-survival Bcl-2 family members, such as Bcl-2 and Bcl-xL, to establish an apoptosis-resistant state (Yosef et al., 2016). Dependence on anti-apoptotic signaling allows them to survive death signals that would normally eliminate damaged cells. Apoptotic resistance provides the molecular basis for senolytic therapies. Extrinsically, senescent cells evade the innate immune system. While the SASP initially attracts NK (natural killer) cells and macrophages, senescent cells can also escape immune surveillance through surface receptor upregulation and protease secretion (Majewska and Krizhanovsky, 2025). Combined with the general decline of immune function during aging, these evasion tactics lead to the pathological accumulation of senescent cells within tissues. The pathological accumulation of senescent cells therefore calls for targeted strategies for their elimination, which will be discussed in the following section.

## 4 Strategies to counteract senescent cell accumulation

The persistence of senescent cells and their resistance to apoptosis present a fundamental challenge for tissue homeostasis. Several strategies have emerged to address their accumulation, each targeting distinct aspects of senescent cell biology. These range from harnessing the body’s innate immune defenses and pharmacological senolytic and senomorphic interventions to mechanically driven elimination through cell competition and the direct reversal of the senescent phenotype *via* cellular rejuvenation. This section examines each of these approaches in turn.

### 4.1 Immune-mediated clearance

The immune system represents the body’s primary natural defense against senescent cell accumulation. Functional immune cells, particularly NK cells and macrophages, are the principal effectors of this process. NK cells detect senescent cells *via* stress ligands upregulated on the cell surface, such as NKG2D ligands, and kill them through perforin-granzyme mediated cytotoxicity (Majewska and Krizhanovsky, 2025). Macrophages contribute through both direct phagocytosis and the secretion of pro-inflammatory cytokines that amplify the broader immune response (Langhi Prata et al., 2018). Despite these defenses, senescent cells are capable of acquiring mechanisms to escape elimination. Overexpression of MMP3 is one common method used to evade NK cell-mediated clearance (Muñoz et al., 2019). Furthermore, senescent cells can upregulate the CD47 (cluster of differentiation 47) inhibitory receptor, providing a ‘do not eat me’ signal that allows them to evade macrophage-mediated phagocytosis (Schloesser et al., 2022). Immunosenescence and inflammaging further worsen this evasion. The continuous SASP inflammatory load, combined with the aging of the immune cells themselves, leads to a gradual decline in overall surveillance capabilities. As the immune system becomes dysfunctional at advanced ages, the ability to naturally clear senescent cells diminishes, resulting in their pathological accumulation within tissues (Majewska and Krizhanovsky, 2025). Since immune cells cannot easily replenish themselves in the elderly, interventions like the administration of carnosine are being explored to boost natural clearance and improve macrophage-mediated destruction of senescent cells.

### 4.2 Senolytics

Senolytics are a class of drugs designed to selectively eliminate senescent cells by targeting the pro-survival pathways that underlie their apoptosis resistance. Evidence suggests that selectively killing these cells improves healthspan in mice and remains effective against age-related diseases and cancer (Baker et al., 2011). The Bcl-2 protein family serves as a primary target in these therapies. Senescent cells often accumulate significant damage and exist on the verge of apoptosis, yet they remain viable due to a heavy dependence on Bcl-2, Bcl-xL, and related anti-apoptotic proteins. Navitoclax (ABT-263) functions as a potent inhibitor of Bcl-2 and Bcl-xL, disrupting this survival dependency to induce apoptosis selectively (Zhu et al., 2016). Similarly, Quercetin (Q), a flavonoid compound, exerts senolytic activity by targeting SCAPs (senescent cell anti-apoptotic pathways), including PI3K/AKT (phosphoinositide 3-kinase/protein kinase B) survival signaling, Bcl-2 family members, and p53/p21/serpine pathways, collectively reducing the anti-apoptotic signaling that senescent cells depend on for survival (Zhu et al., 2015). Tyrosine kinase networks represent a second axis of senescent cell viability. Dasatinib (D), a broad-spectrum tyrosine kinase inhibitor originally developed for leukaemia, exerts senolytic activity primarily in senescent adipocyte progenitors by targeting ephrin receptors and Src kinases, key components of the pro-survival SCAP networks these cells depend on to resist apoptosis (Zhu et al., 2015). The combination of Dasatinib and Quercetin (D + Q) has emerged as the most widely studied and utilized senolytic regimen in clinical trials. Current evaluations are investigating the safety and efficacy of such agents in age-related conditions, including idiopathic pulmonary fibrosis, diabetic chronic kidney disease (Hickson et al., 2019), and Alzheimer’s disease (Gonzales et al., 2022). While the heterogeneity of senescent cell populations presents a challenge for precise targeting, the growing number of clinical trials evaluating senolytics across diverse age-related conditions reflects their significant therapeutic promise.

### 4.3 Senomorphics

In contrast to senolytics, which eliminate senescent cells, senomorphics (also termed senostatics) act by suppressing or modifying the SASP without inducing cell death (Lagoumtzi and Chondrogianni, 2021; Alum et al., 2025). This distinction is therapeutically important. Because senescent cells contribute to development, wound healing, and tumor suppression, indiscriminate elimination may carry unintended consequences. Senomorphics offer a complementary strategy by neutralizing the pathological output of senescent cells while preserving their physiological roles. Several agents have demonstrated senomorphic activity, including rapamycin, which inhibits mTOR (mechanistic target of rapamycin)-driven SASP translation (Laberge et al., 2015); metformin, which suppresses NF-κB activation (Moiseeva et al., 2013); and JAK (Janus kinase) inhibitors, which dampen SASP-driven inflammation (Xu et al., 2015). By targeting the inflammatory and matrix-remodeling components of the SASP rather than the cells themselves, senomorphics may mitigate paracrine senescence and chronic tissue dysfunction while leaving beneficial transient senescent populations intact. By also controlling the microenvironment surrounding senescent cells, senomorphics aim to restore tissue homeostasis and promote tissue regeneration, extending their therapeutic potential beyond mere SASP suppression. Furthermore, senomorphics hold potential as a complementary approach to senolytic therapy in combination treatment regimens, offering a more nuanced modulation of the senescent cell landscape. However, because senomorphics require sustained administration to maintain SASP suppression, long-term safety and the risk of broader immunomodulation remain important considerations.

### 4.4 Mechanical cell competition

Alongside immune-mediated clearance and pharmacological interventions, a mechanically distinct mode of senescent cell elimination has recently emerged from studies of cell competition. This quality control mechanism drives tissue turnover by selecting for the fittest cells while eliminating viable but suboptimal loser cells. While cells often communicate fitness disparities through biochemical signaling or competition for survival factors, a distinct mode known as mechanical cell competition relies instead on physical interactions (Wagstaff et al., 2016; Brás-Pereira and Moreno, 2018; Schoenit et al., 2025). Within this non-autonomous process, mechanical forces such as compressive stress trigger the extrusion and apoptotic death of less fit neighbors, with differential stiffness or compressibility between competing populations emerging as a key determinant of the outcome (Gupta et al., 2024). First established in oncogenic contexts (Levayer et al., 2016; Moreno et al., 2019; Valon et al., 2025), this principle is now being applied to senescent cell elimination.

Mechanical compression has recently been identified as a mechanism for clearing senescent cells (Pan et al., 2026). Although senescent cells are typically resistant to apoptosis and progressively accumulate to disrupt tissue function, those induced by progerin, a mutant form of the nuclear structural protein Lamin A (Kim et al., 2023), become highly susceptible to cell death when co-cultured with healthy neighbors. Normal cells exert active mechanical compression on these senescent neighbors, and this physical input serves as the upstream trigger for their elimination. Contact-dependent mechanical competition initiates a specific signaling cascade in the senescent cells, driven by the upregulation of the stress kinases JNK (c-Jun N-terminal kinase) and p38-MAPK (Mitogen-Activated Protein Kinase) and the tumor suppressor p53, which ultimately triggers their apoptosis.

The mechanical clearance of senescent cells is analogous to mechanical cell competition in the elimination of transformed cells (Wagstaff et al., 2016). A study first demonstrated such a mechanism by showing that normal epithelial cells exert compressive forces to corral suboptimal neighbors, such as those silenced for the polarity tumor-suppressor gene *scribble*. In these transformed cells, compaction acts as a mechanical insult that triggers a mechanotransduction cascade: ROCK activates p38, which elevates p53 to lethal levels and induces apoptosis. Similar mechanical crowding mechanisms lead to the apical extrusion of RasV12-transformed cells (Gupta et al., 2024), supporting mechanical competition as a conserved mechanism for purging suboptimal cells from healthy epithelia.

Interestingly, the mechanical vulnerability of senescent cells appears to be highly context-dependent (Pan et al., 2026). For instance, while senescent cells expressing progerin are cleared by mechanical compression, p16-induced senescent cells do not undergo apoptosis under similar circumstances. This distinction highlights the heterogeneity among different senescent cell populations. These findings also suggest that certain senescent cells may develop resistance to mechanical surveillance in aged tissues, contributing to their pathological accumulation.

### 4.5 Cellular rejuvenation and partial reprogramming

Alongside strategies that eliminate senescent cells, an emerging class of interventions aims to reverse the senescent state, restoring cells to a functionally younger phenotype (Ji et al., 2023; Kureel et al., 2024). This approach is attractive because it avoids the limitations of cell elimination, while promoting the restoration of cellular function in previously senescent cells. At the molecular level, senescence is associated with widespread epigenetic changes, including alterations in DNA methylation, histone modifications, and the three-dimensional organization of chromatin. While partial reprogramming using transiently delivered Yamanaka factors has shown promise in reversing epigenetic aging marks and restoring youthful gene expression, mechanobiological strategies offer a complementary way to achieve rejuvenation by leveraging physical cues to restore cellular function (Kureel et al., 2024; Han et al., 2025).

Low-frequency ultrasound (LFU) is emerging as a non-invasive mechanobiological strategy to rejuvenate senescent cells (Kureel et al., 2025a). LFU creates mechanical pressure waves that subject cells to specific physical stresses. When normal, non-senescent cells are subjected to these waves, the mechanical stress stimulates them to secrete distinct growth-activating factors, including PDGF-BB (Platelet-Derived Growth Factor-BB). Applying these induced secretions to senescent cells can indirectly activate growth *via* paracrine signaling without inducing programmed cell death. Intracellularly, such mechanical stimulation causes a reversal of the senescent phenotype across at least 15 distinct characteristics (Kureel et al., 2025a). Documented changes include the suppression of SASP expression and decreases in β-galactosidase activity, p16, and p21 expression, alongside a measurable increase in telomere length. Mechanical pressure waves also physically alter organelle architecture by inducing the fragmentation of enlarged, dysfunctional mitochondria, and decreasing lysosomal volume. These rejuvenation pathways likely involve the activation of mechanosensitive ion channels, particularly Piezo1, and a subsequent influx of calcium. While the exact mechanisms by which calcium influx contributes to the reversal of the senescent phenotype remain to be fully elucidated, the therapeutic potential is significant.

Changing a cell’s physical microenvironment can also induce partial reprogramming (Kureel et al., 2025b). When aged human fibroblasts are grown on fibronectin-coated micropatterns, they are subjected to sustained lateral confinement (Roy et al., 2020; Sornapudi et al., 2025). Geometric restriction limits cell spreading and reorganization of the cytoskeleton and nuclear architecture. Mechanical confinement shifts gene expression profiles away from an aged, fibroblastic state and induces the formation of stem-cell-like spheroids. Beyond geometric confinement, altering the mechanical stiffness of a 3D microenvironment is another effective strategy for rejuvenation. A study demonstrated that the functional decline of OPCs (oligodendrocyte progenitor cells) is primarily driven by the progressive mechanical stiffening of their extracellular niche (Segel et al., 2019). Culturing aged OPCs on soft polyacrylamide hydrogels designed to mimic the elasticity of youthful tissue rejuvenates the cells and restores their capacities for proliferation and differentiation. Identifying the mechanosensitive ion channel Piezo1 as the key sensor allows researchers to genetically silence or inhibit the channel, effectively ‘deceiving’ aged OPCs into functioning as if they were in a youthful environment. This matrix-driven rejuvenation has been successfully replicated *in vivo*, demonstrating that physical microenvironments are key determinants of cell age states.

## 5 Conclusion and future directions

### 5.1 Conclusion

Apoptotic cells are extruded *via* mechanotransductive pathways, stimulating localized compensatory proliferation in neighboring cells without compromising barrier integrity. In contrast, senescent cells often evade clearance, and their persistent SASP drives ECM stiffening and paracrine senescence, progressively impairing tissue function. While immune-mediated clearance and pharmacological interventions offer routes to reduce senescent cell burden, their efficacy is limited by immune decline and the heterogeneity of senescent cell populations. Mechanical cell competition provides a complementary, cell-intrinsic surveillance mechanism, though its effectiveness varies across senescent cell types. Mechanobiological interventions, such as LFU and geometric confinement, offer a promising complementary approach by leveraging physical cues to partially reprogram senescent cells, reversing their aged phenotype and restoring tissue architecture, although the underlying mechanisms are not yet fully resolved.

### 5.2 Future directions

Several key questions remain regarding how these insights translate into therapeutic strategies. A first set concerns the biological outcome of intervention: whether removing or rejuvenating senescent cells restores tissue mechanical integrity and function, and how formerly senescent or repopulating cells reintegrate into the surrounding mechanical network to ensure long-term tissue stability rather than localized dysfunction. A second set concerns translation: while senolytics are advancing through clinical trials and LFU has reversed senescence markers in aged mouse models, extending any of these strategies safely to diverse human tissues, particularly deep-tissue organs, remains a significant challenge. Translating these strategies into therapy will require new approaches for safely delivering targeted mechanical, chemical, or biological cues to the sites where they are needed.

A further challenge concerns the selectivity of senescent cell elimination. Because senescent cells contribute to embryonic development, wound healing, and tumor suppression (Muñoz-Espín et al., 2013; Demaria et al., 2014), systemic administration of senolytics or other elimination strategies risks depleting beneficial transient senescent populations alongside pathologically accumulated ones. Future therapeutic approaches will therefore need to achieve topical or tissue-targeted delivery, restricting elimination to sites where senescent cells disrupt homeostasis while preserving those engaged in physiological functions. Strategies such as localized drug delivery, tissue-specific senolytic prodrugs activated by SASP-associated markers, and the mechanobiological interventions discussed above may help meet this requirement by acting only on cells within a defined pathological niche.

Together, immune, pharmacological, and mechanobiological strategies offer complementary routes to counteract senescence. Their integration, matched to the heterogeneity of senescent cells across tissues, may ultimately enable the restoration of tissue function in aged or damaged organs.
